# Liver CHOP‐and‐change stress response

**DOI:** 10.1002/hep4.2060

**Published:** 2022-07-28

**Authors:** Pasquale Piccolo, Nicola Brunetti‐Pierri

**Affiliations:** ^1^ Telethon Institute of Genetics and Medicine Pozzuoli Italy; ^2^ Department of Translational Medicine Federico II University of Naples Naples Italy

To the editor,

We read with interest the paper from Lu and colleagues, who investigated the unfolded protein response (UPR) in livers expressing the Z allele of α1‐antitrypsin (Z‐AAT).^[^ ^1^ ^]^ Activation of the UPR in response to hepatic expression of Z‐AAT has been controversial, largely because the available PiZ transgenic mouse was lacking an adequate control (i.e., a mouse transgenic for the wild‐type human AAT). Brantly's laboratory filled this gap by generating brand‐new transgenic mouse models expressing either the human wild‐type AAT or Z‐AAT, which they called PI*M and PI*Z mice, respectively. Lu et al. found partial but sustained activation of UPR in PI*Z mouse livers, but not in Pi*M mice, despite overexpression of wild‐type AAT. In a subset of PI*Z mice with more Z‐AAT storage, they also found up‐regulation of C/EBP homologous protein (CHOP). This finding is consistent with our previous study that reported increased CHOP expression and transcriptional activity in livers from the PiZ transgenic mice and pediatric Pi*ZZ subjects.^[^ ^2^ ^]^ Based on the results of our previous study showing that CHOP binds to the *SERPINA1* (serpin family A member 1) promoter and increases its expression,^[^ ^2^ ^]^ CHOP up‐regulation is also predicted to aggravate the UPR, and therefore potentially exacerbate the liver disease. Therefore, CHOP appears to be involved in a maladaptive response induced by Z‐AAT. This is suggested by normal CHOP expression in a subset of PI*Z mice with reduced Z‐AAT storage.^[^ ^1^ ^]^ Among the three branches of the UPR, Lu et al. found that the activating transcription factor 6 (ATF6) was activated, whereas the other two branches were repressed.^[^ ^1^ ^]^ ATF6 can drive CHOP up‐regulation, but the authors did not mention whether PI*Z mice with CHOP overexpression and larger Z‐AAT storage also have increased ATF6 activation. Moreover, the increased expression of ATF4 found by Lu et al. is puzzling because ATF4 is regulated by the UPR at the translational level rather than transcriptionally, and PERK (PKR‐like endoplasmic reticulum kinase) was found to be repressed.^[^ ^1^ ^]^ In summary, the mechanism underlying CHOP up‐regulation remains elusive.

As a cautionary note, because hepatic Z‐AAT storage has been found to change in aging PiZ mice,^[^ ^3^ ^]^ analysis of UPR in the newly generated transgenic mouse model should be performed across specific age ranges. Despite these limitations, the availability of the new PI*Z and PI*M mouse models is an advancement in the field, and we anticipate they will be used extensively.

## CONFLICT OF INTEREST

NBP consults for Genespire and Pfizer.

## References

[1] Lu Y , Wang LR , Lee J , Mohammad NS , Aranyos AM , Gould C , et al. The unfolded protein response to PI*Z alpha‐1 antitrypsin in human hepatocellular and murine models. Hepatol Commun. 2022 May 27. 10.1002/hep4.1997. [Epub ahead of print] PMC9426387 35621045

[2] Attanasio S , Ferriero R , Gernoux G , De Cegli R , Carissimo A , Nusco E , et al. CHOP and c‐JUN up‐regulate the mutant Z α1‐antitrypsin, exacerbating its aggregation and liver proteotoxicity. J Biol Chem. 2020;295:13213–23. 32723872 10.1074/jbc.RA120.014307 PMC7504927

[3] Piccolo P , Ferriero R , Barbato A , Attanasio S , Monti M , Perna C , et al. Up‐regulation of miR‐34b/c by JNK and FOXO3 protects from liver fibrosis. Proc Natl Acad Sci U S A. 2021;118:e2025242118. 33649241 10.1073/pnas.2025242118 PMC7958360

